# Risk Factors Associated with Loco-Regional Failure after Surgical Resection in Patients with Resectable Pancreatic Cancer

**DOI:** 10.1371/journal.pone.0157196

**Published:** 2016-06-22

**Authors:** Hyun Ju Kim, Woo Jung Lee, Chang Moo Kang, Ho Kyoung Hwang, Seung Min Bang, Si Young Song, Jinsil Seong

**Affiliations:** 1 Department of Radiation Oncology, Yonsei University College of Medicine, Seoul, Korea; 2 Division of Hepatobiliary and Pancreas, Department of Surgery, Yonsei University College of Medicine, Seoul, Korea; 3 Division of Gastroenterology, Department of Internal Medicine, Yonsei University College of Medicine, Seoul, Korea; University of North Carolina School of Medicine, UNITED STATES

## Abstract

**Purpose:**

To evaluate the risk factors associated with loco-regional failure after surgical resection and to identify the subgroup that can obtain benefits from adjuvant radiotherapy (RT).

**Materials and Methods:**

We identified patients treated with surgical resection for resectable pancreatic cancer at Severance hospital between January 1993 and December 2014. Patients who received any neoadjuvant or adjuvant RT were excluded. A total of 175 patients were included. Adjuvant chemotherapy was performed in 107 patients with either a gemcitabine-based regimen (65.4%) or 5-FU based one (34.9%).

**Results:**

The median loco-regional failure-free survival (LRFFS) and overall survival (OS) were 23.9 and 33.6 months, respectively. A recurrence developed in 108 of 175 patients (61.7%). The predominant pattern of the first failure was distant (42.4%) and 47 patients (26.9%) developed local failure as the first site of recurrence. Multivariate analysis identified initial CA 19–9 ≥ 200 U/mL, N1 stage, perineural invasion (PNI), and resection margin as significant independent risk factors for LRFFS. Patients were divided into four groups according to the number of risk factors, including initial CA 19–9, N stage, and PNI. Patients exhibiting two risk factors had 3.2-fold higher loco-regional failure (P < 0.001) and patients with all risk factors showed a 6.5-fold increase (P < 0.001) compared with those with no risk factors. In the analysis for OS, patients with more than two risk factors also had 3.3- to 6-fold higher risk of death with statistical significance.

**Conclusion:**

The results suggest that patients who exhibit more than two risk factors have a higher risk of locoregional failure and death. This subgroup could be benefited by the effective local adjuvant treatment.

## Introduction

Pancreatic cancer is a fatal malignant disease and the fifth leading cause of cancer death in Korea.[1] Surgical resection is thought to be the only curative treatment option for localized pancreatic cancer, but only 10–15% of pancreatic cancer patients are resectable at presentation. Even the median survival is only 10–18 months after surgery alone.[2–5] Most treatment failures occur within 1–2 years after surgery in local recurrence, hepatic metastases, or both.[6,7] Systemic and local adjuvant treatment have been investigated to define their effects. In the Gastrointestinal Tumor Study Group (GITSG) trial,[8,9] median survival for the adjuvant chemoradiotherapy (CRT) group was significantly longer than that observed for the control group (20 months vs. 11 months). However, the European Organization for Research and Treatment of Cancer (EORTC) 40891 trial did not show a statistically significant difference in progression-free survival (PFS) and overall survival (OS) between two arms.[10,11] To clarify the effect of adjuvant CRT, the European Study Group for Pancreatic Cancer (ESPAC-1) trial was performed. Results from the ESPAC-1 trial suggested that adjuvant CRT has a detrimental effect on survival compared with surgery alone.[12,13] Conclusions drawn from the ESPAC-1 trial have raised questions about the efficacy of adjuvant CRT for locally advanced pancreatic cancer, and consequently, current clinical practices tend to exclude adjuvant radiotherapy (RT) from routine adjuvant treatment modalities.

In the clinical setting, however, loco-regional failures as the first recurrence are frequent, resulting in an additional course of RT as a salvage treatment. While consensus regarding the benefit of adjuvant RT is lacking, this study was designed to evaluate the risk factors associated with loco-regional failure after surgical resection and to identify the subgroup of patients who can benefit most from adjuvant RT.

## Materials and Methods

### Study population

We searched to identify all patients treated with surgical resection for pancreatic cancer at Severance hospital from 1993 to 2014. A total of 411 patients were identified and retrospectively reviewed. Among these patients, 236 were excluded from this analysis due to the following reasons: (1) receipt of neoadjuvant CRT before surgical resection. (n = 78); (2) receipt of surgical resection as a palliative aim for stage IV disease (n = 24); (3) receipt of postoperative CRT or RT (n = 39); (4) diagnosed with neuroendocrine tumor (n = 60), intraductal papillary neoplasm (IPMN) without invasiveness (n = 9), and solid pseudopapillary tumor (n = 10); (5) exhibited history of another primary cancer (n = 5); and (6) follow-up after surgical resection was not performed (n = 11). Ultimately, a total of 175 patients were included for this analysis. This study was approved by Institutional review board (IRB) of Yonsei University Health System. The patient records/information was anonymized and de-identified prior to analysis, and informed consent was not obtained from each participants.

### Preoperative assessment and treatment

Pretreatment evaluation included a review of previous medical history, physical examination, laboratory tests and performance status. For staging workup, preoperative computerized tomography, magnetic resonance image, and 18F-fluorodeoxyglucose positron emission tomography-CT were performed.

Resectability was assessed based on imaging studies according to the National Comprehensive Cancer Network (NCCN) classification.[14] Resectable pancreatic cancer was defined as: (1) the absence of distant metastasis, (2) no evidence of tumor invasion to the SMA or celiac axis, and (3) none or less than 180 degree contact with superior mesenteric vein (SMV) or portal vein (PV) without contour irregularity. Vascular invasion was defined as tumor-to-vessel circumferential contiguity, either abutment (≤ 50% of the circumference) or encasement (> 50% of the circumference).

Surgical procedures were composed of pylorus-preserving pancreaticoduodenectomy (PPPD), Whipple operation, or total pancreatectomy for pancreatic head cancer and partial pancreatectomy for a pancreatic body and tail cancer. Surgical margins such as the pancreatic duct, bile duct, retroperitoneal margin, duodenum, or stomach were evaluated grossly and microscopically to elucidate the status of the surgical margins. These surgical margins, with the exception of the retroperitoneal margin, were often evaluated using frozen-section analysis, and if positive, additional resection was performed. The final margin status was reported in the permanent pathology report. Resection status was defined as complete resection with microscopically negative margins (R0), grossly complete resection with microscopically positive margins (R1), and grossly incomplete resection (R2), which is determined by surgeons or on the postoperative imaging studies. Adjuvant chemotherapy was performed in 107 patients (61.1%) with either a gemcitabine-based regimen (70 patients, 65.4%) or 5-FU-based one (37 patients, 34.6%).

The following variables were collected for each patient: patient demographics (age, sex, diabetes mellitus (DM), Eastern Cooperative Oncology Group (ECOG) performance status), tumor characteristics (size, stage, tumor marker, grade, lymphovascular invasion (LVI), perineural invasion (PNI), lymph node (LN) metastasis, perinodal extension (PNE)), and pathologic margin status.

### Statistical analysis

Study endpoints were loco-regional failure-free survival (LRFFS) and OS. Survival duration was calculated from the date of surgical resection to the corresponding event (loco-regional failure, distant metastasis, or death). We performed Pearson’s chi-squared test or Fisher’s exact test to compare categorical variables. The area under the receiver-operating characteristic (ROC) curve (AUC) was used to find optimal cut off value for preoperative CA 19–9 level. The Kaplan–Meier method with log-rank test was used to analyze survival outcomes. Stepwise Cox proportional hazards regression was used to perform a multivariable analysis on prognostic factors for LRFFS and OS (inclusion criteria P < 0.05). All statistical tests were two-sided with significance defined as P < 0.05. The data were analyzed using IBM SPSS software version 20.0 (SPSS Inc., Chicago, IL, USA).

## Results

### Patient characteristics

A total of 175 patients were included in this study. The patient characteristics are summarized in Table 1. The median age was 65 years (range, 34–84 years). ECOG performance status was 0 in 96 patients (54.9%) or 1 in 79 patients (45.1%). Among 122 pancreatic head cancer patients, 101 (58%) received PPPD, 19 (11%) received Whipple’s operation, and 2 (1%) received total pancreatectomy. Fifty-three patients with pancreatic body or tail cancer received partial pancreatectomy. Most of the patients were diagnosed with ductal adenocarcinoma (144 patients), while mucinous carcinoma, acinar cell carcinoma, and IPMN with invasion were diagnosed in 6, 4, and 21 patients, respectively. One-hundred forty three patients were diagnosed with T3 stage based on their pathologic reports. Seven patients were diagnosed with T4 stage, which was initially considered as resectable disease in the preoperative imaging studies. Eighty-seven patients (49.7%) presented with positive nodal status. Resection status was R0 in 160 patients and R1 in 15 patients. The preoperative and postoperative CA 19–9 level ranged from 0.1 to 20,000 U/mL, with a median of 90.3 U/mL and 0.1 to 2060 U/mL, with a median of 12.9 U/mL, respectively. The AUC was 0.588 (95% Confidence interval (CI) 0.504–0.672, P = 0.046) for preoperative CA 19–9 level and 0.541 (95% CI 0.454–0.627, P = 0.362) for postoperative CA 19–9 level. Patients were divided by the cutoff value of 200 U/mL for preoperative CA 19–9 and 40 U/mL for postoperative CA 19–9.

**Table 1:** Patient characteristics.

Characteristics	n	%
Age, mean (range), yr	63.3 (34–84)	
Sex		
Male	108	61.7
Female	67	38.3
ECOG performance status		
0	96	54.9
1	79	45.1
Tumor location		
Head	122	69.7
Body	36	20.6
Tail	17	9.7
Operation name		
PPPD	101	57.7
Whipple operation	19	10.9
Total pancreatectomy	2	1.1
Partial pancreatectomy	53	30.3
Pathology		
Ductal adenoca	144	82.3
Mucinous adenoca	6	3.4
Acinar cell ca	4	2.3
IPMN c invasiveness	21	12.0
T stage		
T1	11	6.3
T2	14	8.0
T3	143	81.7
T4	7	4.0
N stage		
N0	88	50.3
N1	87	49.7
Stage		
I	21	12.0
IIA	67	38.3
IIB	81	46.3
III	6	3.4
Grade		
WD	19	10.9
MD	108	61.7
PD-UD	15	8.5
Unknown	33	18.9
Tumor size		
< 3cm	102	58.3
≥ 3cm	73	41.7
LVI		
No	121	69.1
Yes	54	30.9
PNI		
No	66	37.7
Yes	109	62.3
Resection status		
R0	160	91.4
R1	15	8.6
PNE		
No	149	85.1
Yes	26	14.9
Preoperative CA 19–9		
≥ 200 U/mL	111	63.4
< 200 U/mL	64	36.6
Postoperative CA 19–9		
≥ 40 U/mL	130	74.2
< 40 U/mL	40	22.9
Unknown	5	2.9

Abbreviations: ECOG = Eastern Cooperative Oncology Group; PPPD = pylorus preserving pancreaticoduodenectomy; IPMN = Intraductal papillary mucinous neoplasm; WD = well-differentiated; MD = moderately-differentiated; PD = poorly-differentiated; UD = undifferentiated LVI = lymphovascular invasion; PNI = perineural invasion; RM = resection margin; PNE = perinodal extension.

### Survival outcomes and patterns of failure

With a median follow-up period of 21 months (range 4.0–109.2 months), 108 out of 175 patients (61.7%) developed a recurrence. Median time to first failure was 14 months. The cumulative actuarial rates of any recurrence at 12 and 18 months were 41.6% and 56.5%, respectively. The median LRFFS was 23.9 months and the median OS was 33.6 months with a 5-yr survival rate of 41.5%.

The patterns of failure are summarized in Table 2. Initial sites of failure were composed of local failures (26.9%), regional failures (5.7%), and distant metastasis (42.4%), when counting recurrences in the multiple sites separately. The predominant pattern of the first failure was distant metastasis, mainly in the liver (n = 50), peritoneum (n = 14), lung (n = 10), paraaortic lymph node (n = 3), and bone (n = 2). Among those who had a distant failure, 52 patients experienced distant failure only. Loco-regional failure without distant metastasis as a first site of recurrence developed in 31 patients (17.7%).

**Table 2:** Patterns of failure.

Failure pattern		No (%)
Local		47 (26.9)
	Local only	27 (15.4)
	Local & regional	2 (1.2)
	Local & distant	16 (9.1)
	Local & regional & distant	2 (1.2)
Regional		11 (6.3)
	Regional only	3 (1.7)
	Regional and distant	4 (0.6)
Distant		74 (42.4)
	Distant only	52 (29.7)

### Risk factors related to LRRF and OS

**Fig 1:**
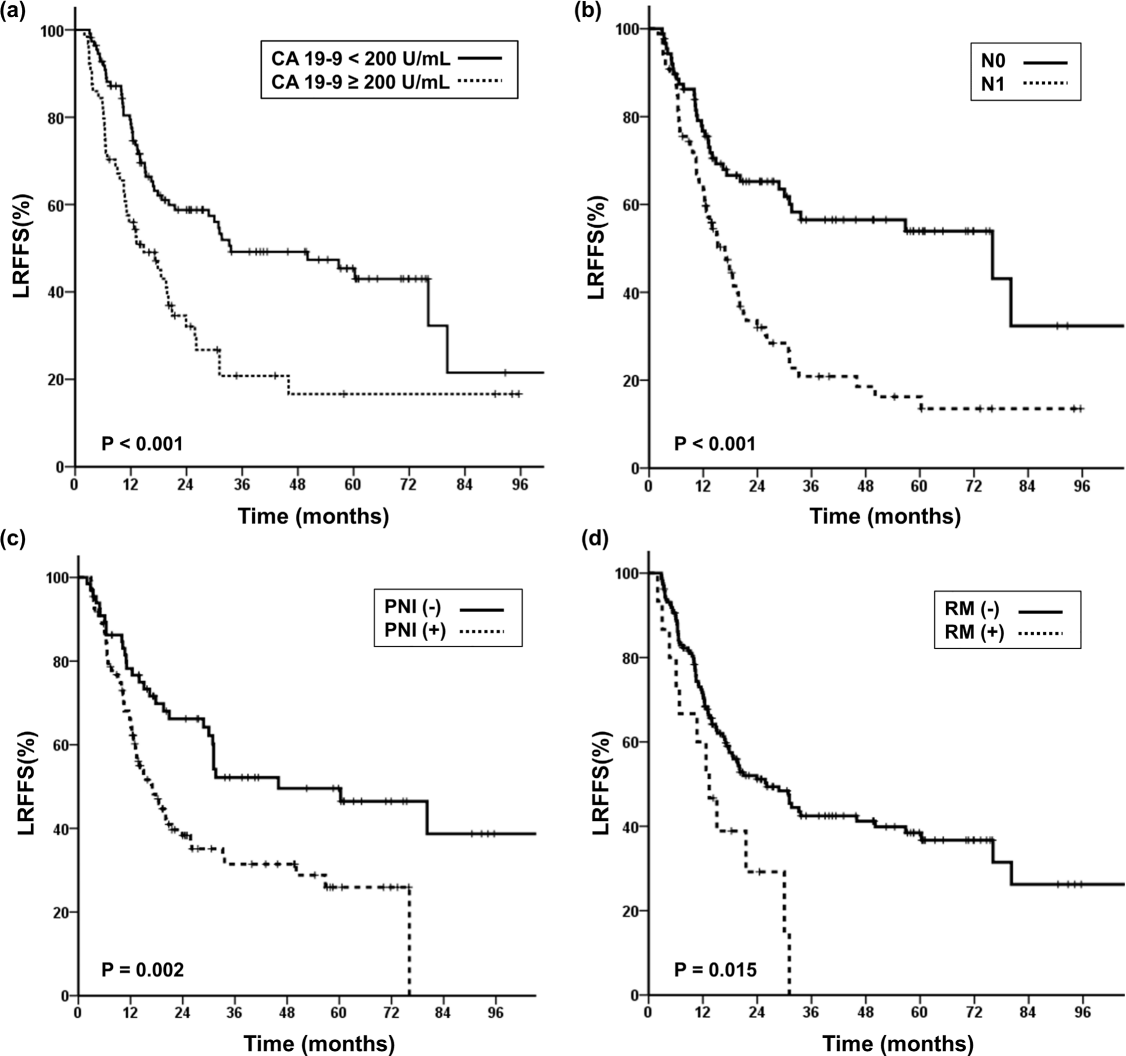
Prognostic factors associated with Loco-regional failure-free survival (LRFFS). Kaplan-Meier curves of Loco-regional failure-free survival (LRFFS) stratified by (a) Initial CA 19–9, (b) N stage, (c) Perineural invasion (PNI) and (d) Resection margin.

We analyzed the prognostic factors related to LRFFS and OS (Table 3). In the univariate analysis, ECOG performance status, preoperative CA 19–9 (≥ 200 U/mL), postoperative CA 19–9 (≥ 40 U/mL), N stage, AJCC stage, LVI, PNI, resection margin, and PNE were associated with LRFFS with statistical significance. Among these, preoperative CA 19–9, N stage, PNI, and resection margin were analyzed as independent prognostic factors for LRFFS in multivariate analysis (Fig 1). Then, all patients were divided into four groups according to the number of risk factors they possessed among these three factors: preoperative CA 19–9 (≥ 200 U/mL), N stage, and PNI. Because positive resection margin is already known as a prognostic factor that requires adjuvant RT, resection margin was not counted as a risk factor in the following analysis. Group 0 was defined as patients who did not exhibit any risk factors, group 1 was defined as those who had one risk factor, and group 2 was defined as those who possessed two risk factors. Group 3 was defined as patients who displayed all three risk factors.

**Table 3:** Prognostic factors for locoregional failure-free survival (LRFFS) and overall survival (OS).

	**LRFFS**					
		**UVA**			**MVA**	
**Characteristic**	**HR**	**95% CI**	**P**	**HR**	**95% CI**	**P**
Age, years (< 65 vs. ≥65)	1.258	0.85–1.87	0.258			
Sex (male vs. female)	0.917	0.61–1.38	0.674			
Diabetes mellitus (no vs. yes)	1.014	0.67–1.53	0.947			
ECOG performance status (0 vs. 1)	1.58	1.06–2.35	0.023	1.315	0.86–2.01	0.206
Preop CA 19–9 (< 200 U/mL vs. ≥200 U/mL)	2.02	1.35–3.02	<0.001	1.738	1.04–2.92	0.037
Postop CA 19–9 (< 40 U/mL vs. ≥40 U/mL)	1.789	1.15–2.79	0.01	0.996	0.57–1.73	0.99
Tumor size (< 3cm vs. ≥3cm)	0.756	0.50–1.14	0.185			
T stage (T1-2 vs. T3-4)	1.932	1.03–3.64	0.041	1.048	0.48–2.31	0.908
N stage (N0 vs. N1)	2.412	1.60–3.64	<0.001	1.661	1.03–2.69	0.04
Grade (WD-MD vs. PD-UD)	0.953	0.48–1.90	0.891			
LVI (no vs. yes)	1.643	1.09–2.48	0.018	1.134	0.72–1.79	0.589
PNI (no vs. yes)	1.975	1.28–3.06	0.002	1.813	1.09–3.01	0.021
Resection margin (R0 vs. R1)	2.101	1.14–3.87	0.017	1.99	1.04–3.81	0.037
PNE (no vs. yes)	2.547	1.53–4.23	<0.001	1.57	0.88–2.82	0.13
Chemotherapy (no vs. yes)	1.053	0.70–1.59	0.805			
	**OS**					
		**UVA**			**MVA**	
**Characteristic**	**HR**	**95% CI**	**P**	**HR**	**95% CI**	**P**
Age, years (< 65 vs. ≥65)	1.648	1.06–2.56	0.027	1.489	0.94–2.37	0.093
Sex (male vs. female)	0.93	0.60–1.45	0.749			
Diabetes mellitus (no vs. yes)	0.947	0.60–1.50	0.816			
ECOG performance status (0 vs. 1)	1.373	0.89–2.13	0.157			
Preop CA 19–9 (< 200 U/mL vs. ≥200 U/mL)	1.891	1.22–2.94	0.005	1.375	0.77–2.45	0.28
Postop CA 19–9 (< 40 U/mL vs. ≥40 U/mL)	2.034	1.26–3.29	0.004	1.289	0.72–2.33	0.398
Tumor size (< 3cm vs. ≥3cm)	0.829	0.53–1.30	0.416			
T stage (T1-2 vs. T3-4)	1.658	0.87–3.16	0.125			
N stage (N0 vs. N1)	2.439	1.55–3.83	<0.001	1.897	1.15–3.13	0.012
Grade (WD-MD vs. PD-UD)	0.943	0.43–2.06	0.883			
LVI (no vs. yes)	1.911	1.21–3.02	0.006	1.413	0.85–2.34	0.181
PNI (no vs. yes)	2.011	1.25–3.25	0.004	1.838	1.08–3.12	0.024
Resection margin (R0 vs. R1)	2.305	1.18–4.51	0.015	2.113	1.02–4.36	0.043
PNE (no vs. yes)	2.165	1.20–3.92	0.011	1.13	0.57–2.26	0.729
Chemotherapy (no vs. yes)	0.978	0.62–1.53	0.921			

**Fig 2:**
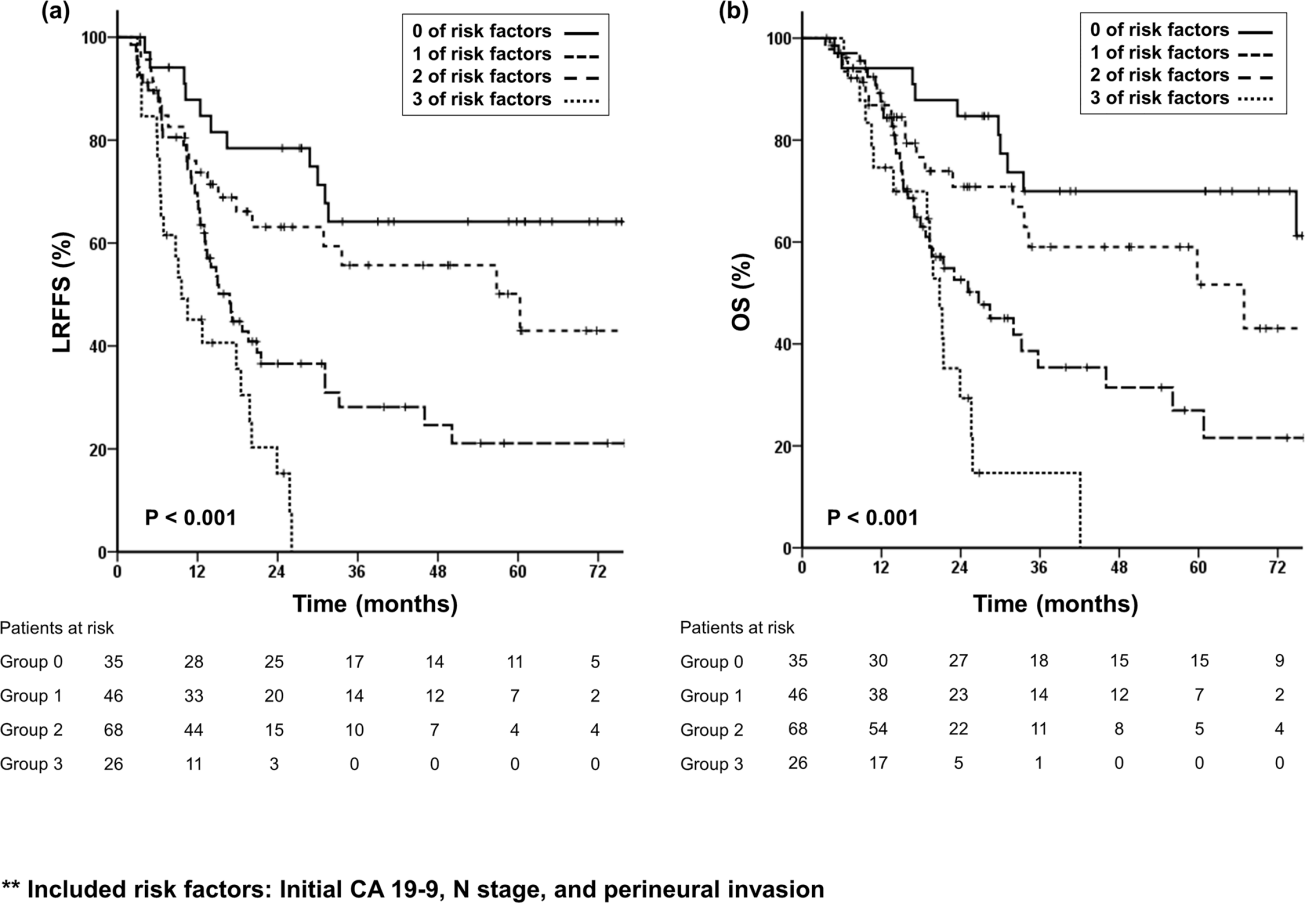
The number of risk factors is associated with locoregional failure-free survival (LRFFS) and overall survival (OS). Kaplan-Meier curves of (a) Locoregional failure-free survival (LRFFS) and (b) Overall survival (OS) according to the number of risk factors.

LRFFS showed statistically significant differences according to the number of risk factors that patients exhibited (Fig 2A). No significant difference was shown between groups 0 and 1 (Hazard ratio (HR) 1.73, P = 0.133), while groups 2 and 3 showed significant differences in LRFFS compared with group 0. Patients in group 2 had a 3.2-fold higher risk of loco-regional failure (P < 0.001, 95% CI 1.66–6.06) and patients in group 3 had a 6.5-fold higher risk (P < 0.001, 95% CI 3.12–13.45) compared with those in group 0.

For OS, age (< 65 years), preoperative CA 19–9 (≥ 200 U/mL), postoperative CA 19–9 (≥ 40 U/mL), N stage, AJCC stage, LVI, PNI, resection margin, and PNE were shown to be prognostic factors with statistical significance in univariate analysis. In multivariate analysis, N stage, PNI, and resection margin were independant prognostic factors associated with survival, which were same as in LRFFS.

We investigated whether the same groups as defined by the number of risk factors used in the LRFFS analysis would align with OS. Our data demonstrated that OS also appeared to decrease sequentially in accordance with the higher number of risk factors (Fig 2B). Patients in group 2 had a 3.3-fold higher risk for death (P = 0.001). Group 3, consisted of those who displayed all three risk factors, showed a 6-fold higher risk for death compared with group 0 (P < 0.001). However, patients in group 1 showed a 2-fold higher tendency for death (P = 0.109).

## Discussion

In this study, we analyzed the patterns of first failure in patients with resected pancreatic cancer and investigated risk factors associated with LRFFS and OS. The purpose of this study was to identify the subgroup that can benefit most from adjuvant RT. Among the patient demographics and clinical-pathological factors, preoperative CA 19–9 ≥ 200 U/mL, N stage, and PNI were significant prognostic factors associated with loco-regional failure. LRFFS and OS showed significant differences between groups defined by the number of these risk factors they possessed. Patients with more than two risk factors had a 3.2- to 6.5-fold higher risk of loco-regional failure, as well as a 3.3- to 6-fold higher risk of death with statistical significance. Thus, we suggest that adjuvant radiotherapy could be beneficial for patients who have two risk factors or more.

Our results are concordant with other prior data that demonstrated distant metastases as the primary pattern of failure in this patient population. In our study, about 42% of patients experienced distant metastasis and loco-regional failures occurred in 33.2% of patients. Similarly, in the study from Johns Hopkins hospital, 68.9% of patients developed distant disease and 31% of patients developed local failure as the first recurrence.[15] The experimental arm of the CONKO-001 trial, which was assigned to receive gemcitabine after resection, experienced distant metastasis rates of 56% and local failure rates of 34%.[16]

In the current practice of surgical resection followed by adjuvant chemotherapy, 60–70% of patients still experienced recurrences after surgical resection.[15,16] To improve outcomes, several studies have been performed to find the optimal adjuvant treatment. In the GITSG trial, adjuvant CRT showed a significant benefit on survival; however, this study has been criticized for the small number of patients evaluated (n = 43).[8,9] The EORTC 40891 phase III trial was performed in Europe to investigate the effect of adjuvant RT with 5-FU. According to this study, those who underwent adjuvant CRT had no significant improvement in survival compared with those who received surgery alone.[10,11] In line with the GITSG and EORTC trials, the ESPAC-1 trial was performed to demonstrate the effect of adjuvant CRT. Results from the ESPAC-1 trial showed a deleterious effect of adjuvant CRT on survival (median 15.9 vs. 17.9 months, P = 0.05), whereas adjuvant chemotherapy had a significant survival benefit (median 20.1 vs. 15.5 months, P = 0.009).[12,13] Based on the ESPAC-1 trial, current practice was changed to omit adjuvant RT for patients with resected pancreatic cancer. However, the ESPAC-1 trial has also been criticized due to its study design and quality control.[17,18] The 2×2 factorial design is inappropriate to make a decisive conclusion about the effect of adjuvant CRT. Indeed, 33% of those assigned to receive chemotherapy did not complete their chemotherapy regimen. Moreover, 17% of the patients did not receive chemotherapy at all. With respect to RT techniques, 40 Gy given in a split-course is no longer used in current practice. Radiation field, dosimetry specifications, and overall quality assurance were not described.

To clarify the benefit of adjuvant CRT, several retrospective studies were performed.[19–23] A retrospective study from the Mayo Clinic that included 472 patients investigated outcomes after surgery alone versus surgery plus adjuvant CRT. OS was better in patients who received adjuvant CRT (median OS, 25.2 vs. 19.2 months, P = 0.001).[19] Another large collaborative study from the Johns Hopkins Hospital and Mayo Clinic [20] showed a survival benefit from adjuvant 5-FU-based CRT compared with surgery alone (median 21.1 vs. 15.5 months, P < 0.001). In an analysis of the National Cancer Data Base (NCDB), 6165 patients with pT1-3N0-1M0 pancreatic adenocarcinoma were categorized into two groups, namely adjuvant CRT and adjuvant chemotherapy, and the outcome was assessed to provide a modern estimate of comparative effectiveness. Adjuvant CRT was independently associated with improved OS compared with adjuvant chemotherapy (median 22.3 vs. 20.0 months, P = 0.001).[23] The benefit of adjuvant RT with modern techniques is still controversial and the subgroup that can benefit most from this approach is not well defined. A well-designed randomized controlled study with modern techniques will be necessary to confirm the efficacy of adjuvant CRT in this population. The RTOG 0848 phase II/III trial is now ongoing to compare gemcitabine together with or without erlotinib hydrochloride and/or radiation therapy after surgery and the result is pending.

In this study, initial CA 19–9 ≥ 200 U/mL, N status, PNI, and resection margin were independent risk factors correlated with LRFFS. Preoperative CA 19–9 level has been proven to be an important predictor of recurrence and survival in patients treated with surgical resection through previous studies.[24–26] In our previous study, CA 19–9 level was demonstrated to be an important prognostic factor associated with survival in unresectable or borderline-resectable pancreatic cancer patients treated with CRT.[27] Lymph node metastasis is also one of the most significant predictive factors on survival.[13,26] Hsu et al.[20] analyzed pathologic N1 status as important factor associated with overall survival, and also demonstrated that resection margin was a significant factor predicting survival.

We excluded patients who received neoadjuvant or adjuvant RT with an intention to eliminate any possible effect of radiotherapy on loco-regional failure, which might be a major confounding factor. The patients who received adjuvant RT due to positive resection margin were also excluded, which might have induced a selection bias. Fifteen patients with positive resection margins included in this analysis were those who refused adjuvant RT despite their positive resection margin status. Local recurrence was significantly higher in those patients, 9 out of 15 patients (60%) experienced local recurrence. In this study, resection margin status was also revealed as an independent prognostic factor associated with LRFFS as well as OS.

This study is limited by the nature of its retrospective design. Data for the length of hospital stay, postoperative recovery, and surgical complications were not available for some patients, which might have been a significant influence in deciding their consequent adjuvant treatment. The selection bias may occur in the process of excluding patients treated with adjuvant RT, which was inevitable. The administration of adjuvant chemotherapy and the adjuvant chemotherapy regimens were not homogenous because they were administered according to the clinician.

In conclusion, patients with more than two risk factors among initial CA 19–9 ≥ 200 U/mL, N1 stage, and PNI have a 3.2- to 6.5-fold higher risk of loco-regional failure, as well as a 3.3- to 6-fold higher risk of death. We suggest that adjuvant local radiotherapy is beneficial to improve the survival outcome of patients with more than two risk factors. To clarify the effect of adjuvant RT, future well-designed randomized controlled studies are needed.
